# Breath can discriminate tuberculosis from other lower respiratory illness in children

**DOI:** 10.1038/s41598-021-80970-w

**Published:** 2021-02-01

**Authors:** Carly A. Bobak, Lili Kang, Lesley Workman, Lindy Bateman, Mohammad S. Khan, Margaretha Prins, Lloyd May, Flavio A. Franchina, Cynthia Baard, Mark P. Nicol, Heather J. Zar, Jane E. Hill

**Affiliations:** 1 Thayer School of Engineering, Dartmouth College, Hanover, NH USA; 2 Geisel School of Medicine, Dartmouth College, Hanover, NH USA; 3 Department of Pediatrics and Child Health, MRC Unit on Child and Adolescent Health, University of Cape Town and Red Cross War Memorial Children’s Hospital, Cape Town, South Africa; 4 Molecular Systems, Organic and Biological Analytical Chemistry Group, University of Liège, Liège, Belgium; 5 Division of Medical Microbiology and Institute for Infectious Diseases and Molecular Medicine, University of Cape Town, Cape Town, South Africa; 6 School of Biomedical Sciences, University of Western Australia, Perth, Australia

**Keywords:** Machine learning, Cheminformatics, Small molecules, Diagnostic markers, Tuberculosis, Paediatric research

## Abstract

Pediatric tuberculosis (TB) remains a global health crisis. Despite progress, pediatric patients remain difficult to diagnose, with approximately half of all childhood TB patients lacking bacterial confirmation. In this pilot study (n = 31), we identify a 4-compound breathprint and subsequent machine learning model that accurately classifies children with confirmed TB (n = 10) from children with another lower respiratory tract infection (LRTI) (n = 10) with a sensitivity of 80% and specificity of 100% observed across cross validation folds. Importantly, we demonstrate that the breathprint identified an additional nine of eleven patients who had unconfirmed clinical TB and whose symptoms improved while treated for TB. While more work is necessary to validate the utility of using patient breath to diagnose pediatric TB, it shows promise as a triage instrument or paired as part of an aggregate diagnostic scheme.

## Introduction

Tuberculosis (TB) is a leading cause of childhood mortality, with an estimated one million cases and 250,000 deaths reported each year^1–3^. While an accurate appraisal of underdiagnosis for childhood TB is unavailable, modelling estimates indicate that only 30% of childhood TB cases are diagnosed and notified^2^. Diagnosing pediatric TB is challenging. Children present with non-specific clinical symptoms, the available tests have poor sensitivity in this population, and there is often a lack of expertise and infrastructure available to obtain microbiologic confirmation in young children^1,4,5^. Even in well-resourced areas, the diagnostic yield from microbiological specimens is sub-optimal, with approximately 50% of pediatric patients diagnosed with TB not having bacterial confirmation^5^. Children co-infected with Human Immunodeficiency Virus (HIV) are particularly challenging to diagnose as the clinical presentation of TB is non-specific and immune deficiency often modifies the clinical presentation of TB disease^6^. The sensitivity of cartridge-based nucleic acid amplification assays for *Mycobacterium tuberculosis*, such as Xpert MTB/RIF and Xpert MTB/RIF Ultra (Xpert, Cepheid, Sunnyvale, California), in children is low: 62% and 75.2% respectively. Moreover, such specimens often rely on induced sputum, which, while safe, cheap and well tolerated, may be difficult to do in children in health care facilities especially in low and middle income country settings^7,8^. Moreover, induced sputum relies on trained personnel and laboratory infrastructure to test samples^8^.

National Institutes of Health consensus guidelines for diagnostic studies of TB in children, classify children as having ‘confirmed TB’ (positive culture or Xpert MTB/RIF test for *M. tuberculosis*), ‘unconfirmed TB’ (negative microbiological results, but clinically diagnosed and treated for TB) or ‘unlikely TB’ (negative microbiologic investigations, not clinically diagnosed with PTB and improvement in the absence of TB treatment)^9^. Consistently, approximately half of pediatric patients diagnosed with TB fall into the unconfirmed TB category in studies^4, 5^. There is a clear need for improved diagnostics for children, particularly for those in the unconfirmed TB category^3,10^.

Previously, we and others have demonstrated that exhaled breath can be collected, analyzed, and mined for putative biomarkers for TB in adults^10–26^. The APOPO non-profit organization have demonstrated that African giant pouched rats can be trained to ‘sniff’ TB, resulting in a substantial increase in case detections, even in children^27–41^. In this pilot study we investigate whether exhaled breath from children has diagnostic utility in a pilot South African study of pediatric patients diagnosed with confirmed TB, unconfirmed TB, or unlikely TB.

## Results and discussion

### Study population

Of the 31 children recruited, 10 (32.3%) were confirmed to have TB disease, 11 (35.4%) had unconfirmed TB, and 10 (32.3%) were unlikely to have TB disease. All confirmed TB patients had at least one positive culture or positive Xpert MTB/RIF test result. Two of these patients had TB confirmed on a cervical lymph node aspirate. Unlikely TB patients had both a negative culture and Xpert MTB/RIF test, and were diagnosed with a lower respiratory tract infection (LRTI) not due to TB. The mean (SD) age of the children was 6 (3.1) years and did not differ significantly by TB category (*p *value 0.874). Overall, 16 (52%) of patients were male, 8 (26%) were underweight for their age, and 7 were stunted (23%). These compositions did not vary significantly by TB category (*p *values 0.997, 0.305, and 0.507 respectively). There was 1 (10%) HIV-infected child in the confirmed TB category, 0 in the unconfirmed TB category and 2 (20%) in the unlikely TB category (*p* value 0.301*)*. A positive tuberculin skin test occurred in 14 children (45.2%); this differed across TB category wherein 60% of confirmed TB patients, 73% of unconfirmed TB patients, and 0% of unlikely TB patients had positive tuberculin skin tests. Demographic and clinical characteristics of the study groups are shown in Table 1. More details about each patient can be found in Supplementary Table 1.

**Table 1:** Demographic and clinical characteristics across the TB study group.

		Confirmed TB (n = 10)	Unconfirmed TB (n = 11)	Unlikely TB (n = 10)	Overall (n = 31)
Age (years)	Mean (SD)	6.3 (3.5)	5.64 (3.4)	6.2 (2.5)	6.0 (3.1)
Sex	Male	6 (60%)	5 (45%)	5 (50%)	16 (52%)
Weight-for-age	Underweight	1 (10%)	3 (27.3%)	4 (40%)	8 (25.8%)
Height-for-age	Stunted	1 (10%)	3 (27.3%)	3 (30%)	7 (22.6%)
HIV-infected	Positive	1 (10%)	0 (0%)	2 (20%)	3 (9.7%)
Tuberculin skin test	Positive	6 (60%)	8 (72.7%)	0 (0%)	14 (45.2%)
	Negative	2 (20%)	1 (9.1%)	10 (100%)	13 (41.9%)
	Missing	2 (20%)	2 (18.2%)	0 (0%)	4 (12.9%)

### Four compounds in breath characterize children with a confirmed TB diagnosis from unlikely TB patients with an alternate lower respiratory tract infection

A Boruta feature selection algorithm was used to identify all relevant compounds for the task of classifying confirmed TB from unlikely TB patients. Four compounds were consistently ranked as more important than shadow features over 84 iterations (results shown in Supplementary Fig. 1). These analytes comprise: decane and 4-methyloctane (identities confirmed by comparing both retention indices and mass spectra with authentic standards) as well as two analytes (labelled Analyte A and B), whose retention times and mass spectra are consistent, but for which we could not find a suitable analytical standard for mass spectral confirmation. Chromatographic and mass spectral information on the four compounds is found in Supplementary Tables 2 and 3 and Supplementary Figs. 6–10.

**Figure 1:**
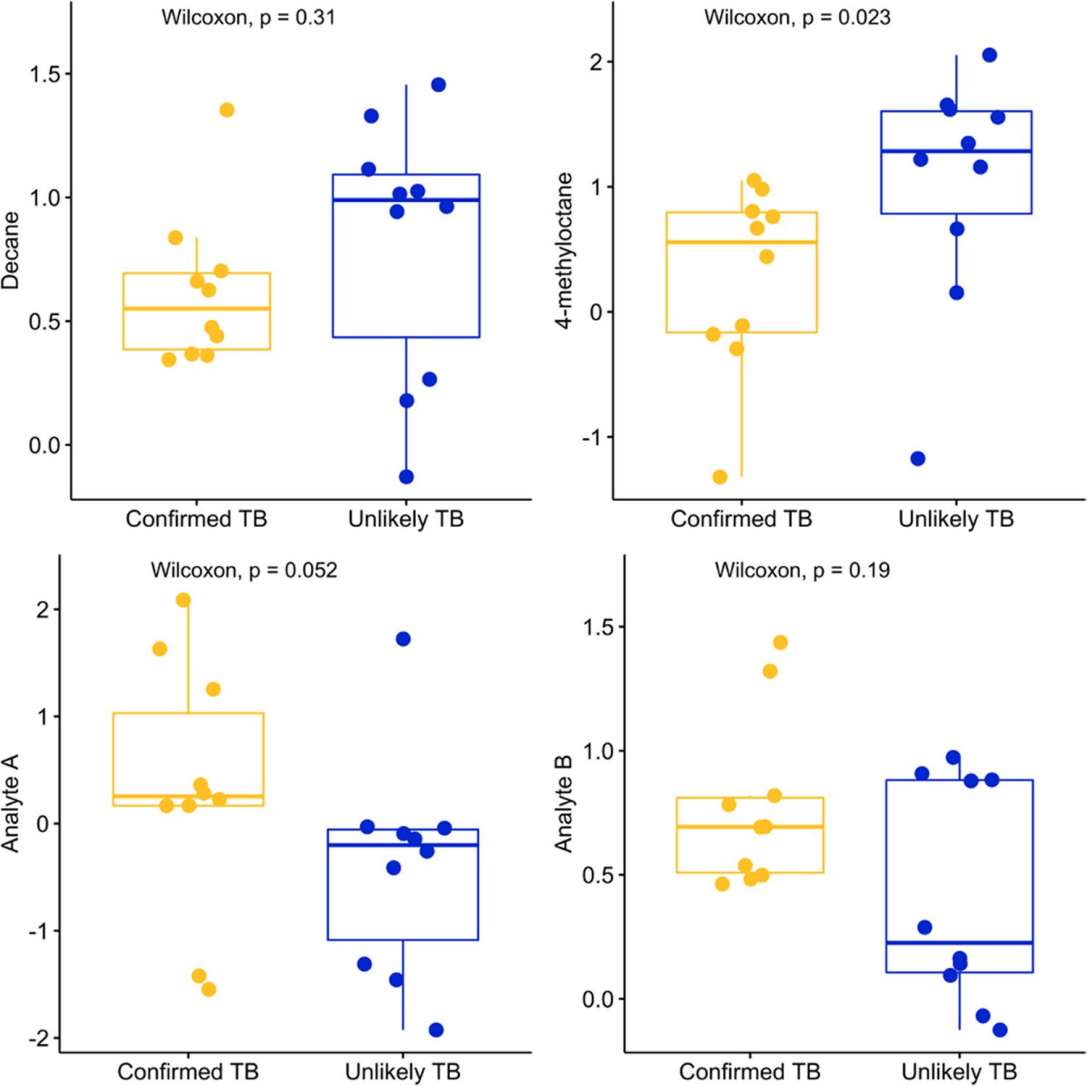
The distribution of the mean centered and normalized peak area of each of the four compounds selected in the breathprint across confirmed TB and unlikely TB patients. For each compound, the median observed peak area between the two groups is different, indicating univariate differences which may contribute to the discrimination of confirmed TB patients from unlikely TB patients. Boxplots show the quartiles of the data (first line is the first quartile, midline is the median, third line is the third quartile) where whiskers represent 1.5 $\times$ IQR (inter-quartile range). The distribution across all three TB groups is shown in Supplementary Fig. 2. Figure created in R^73^ using ‘ggplot2’^84^ and ‘ggpubr’^85^.

The distribution of the each breathprint compound across confirmed TB and unlikely TB groups using normalized chromatographic peak area is shown in Fig. 1. Despite the small sample size, Analyte A and 4-methyloctane are statistically significant at a cut off of α = 0.1 (*p* = 0.052 and *p* = 0.023, respectively). Decane and Analyte B did not reach statistical significance, however, different medians across both groups are observable. The unlikely TB group encapsulates a spectrum of non-TB lower respiratory tract infection (LRTI) cases, therefore it is reasonable to expect greater heterogeneity. The totality of these results supports the hypothesis that a multivariate signature of breath compounds is necessary and should be a focus of investigation in follow up studies.

Machine learning procedures allow us to build a predictive model to evaluate how accurately the four compounds categorized patients according to TB status. Here, we evaluated a random forest model and a support vector machine model with a polynomial kernel using the four features selected with the Boruta algorithm^12,42,43^. Random forest performed best and is discussed further. The SVM model, while complementary to the results from random forest, had slightly lower performance (see Supplementary Table 4 and Supplementary Fig. 3).

**Figure 2:**
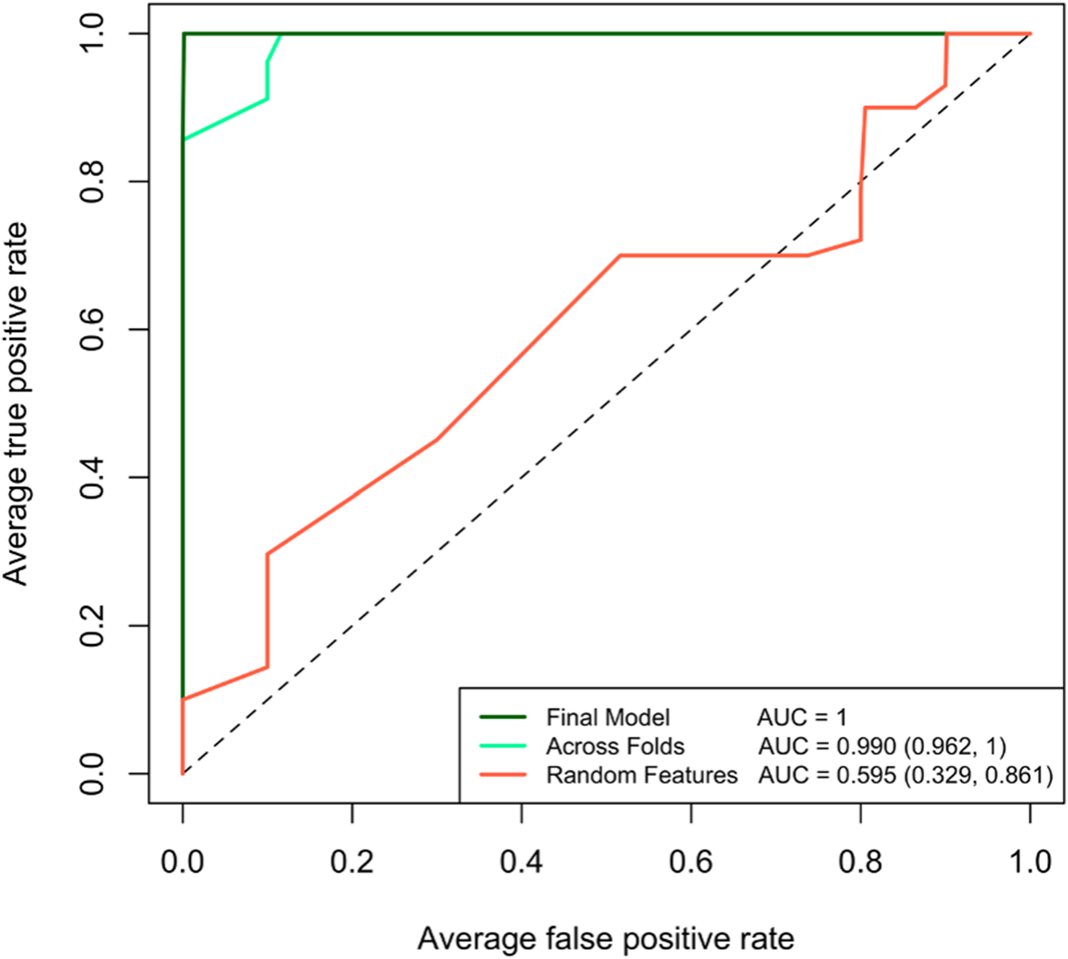
The receiver operating characteristic curves from the random forest model used to classify confirmed TB from unlikely TB patients. The final model demonstrates perfect classification but is almost certainly overfit to the data. The AUC observed across folds using the identified breathprint is 0.99, demonstrating very good sensitivity and specificity across all folds of the data. In comparison, a randomly selected 4-compound breathprint only demonstrated an AUC of 0.595 across cross validation folds of the data. Figure created in R^73^.

The observed area under the receivor operating curve across cross validation folds using the random forest model, shown in Fig. 2, was 0.99 with a 95% confidence interval of (0.961, 1). To better interpret the area under the receiver operating characteristic curve, we selected four compounds in the data at random and repeated the model building process. The observed area under the receiving operating curve across cross validation folds with four randomly selected compounds was 0.595 (0.329, 0.861), clearly demonstrating the utility of the Boruta-selected signature. The WHO’s guidelines for a TB triage test recommend a specificity of 75% and a sensitivity equal to that of Xpert MTB/RIF (62% in children)^44,45^. Across cross validation folds, we observed an accuracy 90%, sensitivity of 80% and specificity of 100%. More performance statistics for the final model can be found in Supplementary Table 5. These data suggest that the four compound candidate biomarkers for pediatric subjects could be a promising route to investigate further.

Biologically, there is evidence to suggest the four compound breathprint characterizes TB. Decane in breath has been previously associated with TB in adults, and is also linked to isoniazid resistance in *Mtb*^16,46^. Both decane and 4-methyloctane have also been identified as characterizing in the breath of adults with asthma, chronic obstructive pulmonary disease (COPD), or lung cancer compared to controls^47–52^. More research in a larger population will be necessary to quantify the differences in production of 4-methyloctane and decane across respiratory diseases.

Analyte A is a nitrogen-containing cyclic compound most likely to be a benzamide derivative. Benzamide derivatives have been detected in the breath of emphysema patients and smokers^53,54^ and are often studied as possible inhibitors of *M. tuberculosis*^55,56^*.* Analyte B is an eleven-carbon alkene that is likely to be branched. Alkenes have been detected in the breath of patients with lung cancer^57^. To identify the precise molecular formula of analytes A and B, follow up studies will need to utilize a high-resolution mass-spectral instrument or equivalent. For more information about the chemical identity of analytes A and B, see Supplementary Table 3 and Supplementary Figs. 9,10.

### The four compound breathprint classifies unconfirmed TB patients

Unconfirmed TB patients are suspected as having TB but do not have a positive culture or Xpert MTB/RIF test result. All unconfirmed TB patients in this study demonstrated improvement of symptoms and weight gain in response to TB treatment. Boxplots comparing the distribution of the mean centered and normalized peak area across the four analytes for each of the three TB categories is shown in Supplementary Fig. 2.

**Figure 3:**
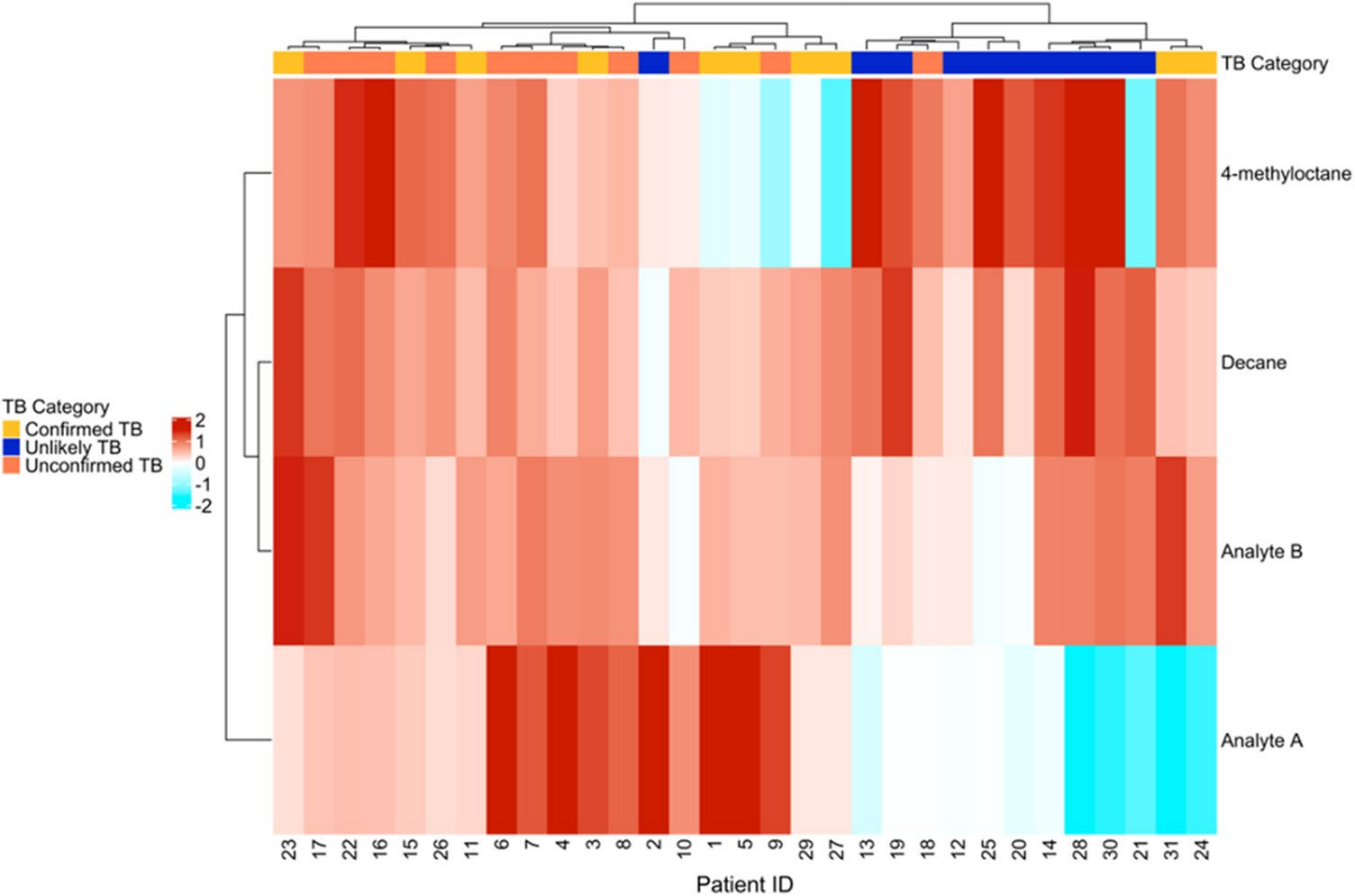
A dendrogram and heatmap demonstrating the unsupervised clustering of patients using the 4-compound breathprint. The annotation bar along the dendrogram indicates TB category. The heatmap shows the normalized peak area for each compound. Red indicates above average peak area, and blue indicates below average peak area. Figure created in R^73^ using ‘ComplexHeatmap’^86^.

Using the four compound breathprint generated by the Boruta approach, the unconfirmed TB cases cluster closely to the confirmed TB cases and also share a similar pattern of relative compound presence in the breath samples (Fig. 3). Overall, 10 of the 11 unconfirmed TB patients cluster closely with the confirmed TB group and away from the unlikely TB patients. Patient 18 is the only unconfirmed patient that did not cluster closely with the confirmed TB group. Both confirmed TB patients 31 and 24 also cluster away from the main TB disease group, which may reflect the occurrence of extra-pulmonary TB (patient 31 had Spinal TB and patient 24 had lymph node TB; see Supplementary Table 1 for more clinical information about the patients). Notably, we observe no clustering by HIV status; patients 20, 19, and 27 are all HIV positive and cluster primarily with their likely TB category participants as opposed to each other. This suggests that our breathprint may be effective for patients with HIV co-infection. A larger cohort for further study will inform further interpretations with less speculation.

**Figure 4:**
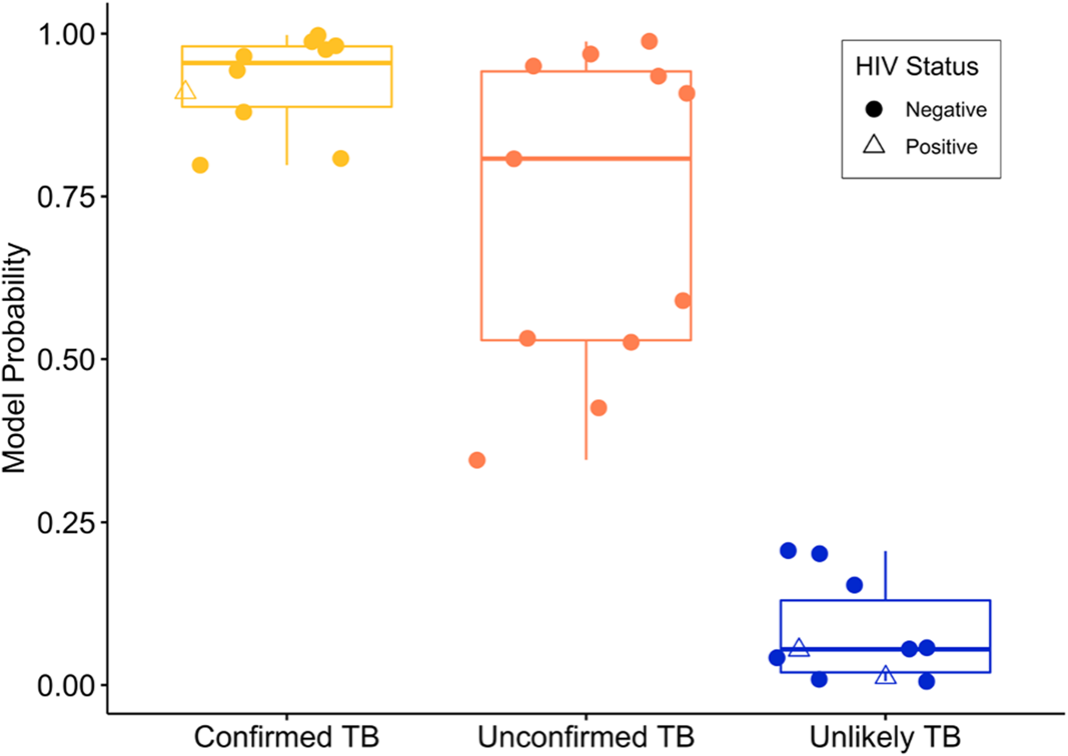
The output probabilities that each patient has TB disease from the random forest classifier across the TB categories. Patients with a probability of over 50% are assigned a label of having TB disease. Despite two unconfirmed TB patients having probabilities below 50%, there is clear differentiation in model probabilities between the unconfirmed and unlikely TB groups. Boxplots show the quartiles of the data (first line is the first quartile, midline is the median, third line is the third quartile) where whiskers represent 1.5 $\times$ IQR (inter-quartile range). Figure created in R^73^ using ‘ggplot2’^84^ and ‘ggpubr’^85^.

We evaluated how well the classification models described for confirmed TB and unlikely TB reference groups might predict the TB status of the unconfirmed TB group (Fig. 4). The random forest classifier correctly predicted 9 of the 11 unconfirmed TB patients (the results for the equivalent SVM analysis which also correctly classifies 9 of the unconfirmed TB patients are given in Supplementary Fig. 4). Six of those patients had probabilities indistinguishable from the confirmed TB cases. Despite two cases having model probabilities below 50%, there is obvious differentiation between the unconfirmed and unlikely TB categories. Specifically, the minimum probability among unconfirmed TB patients was 0.344, while the highest probability among unlikely patients is 0.242, indicating a TB cut-off between these two values exists that would perfectly classify every patient.

The clinical sensitivity of Xpert MTB/RIF is low in unconfirmed pediatric TB patients. If clinical diagnosis is considered the reference standard, the sensitivity of Xpert MTB/RIF in culture-negative samples from pediatric patients ranges from 4 to 15%^58^. Using the proposed 4-compound breathprint, the sensitivity among clinically diagnosed, but microbiologically-negative, pediatric patients is 82% (using a model probability cut-off value of 50%). Achieving 100% sensitivity and specificity is possible in this group if a model cut-off between 25 and 34% is used. Importantly, while confirmation status of patients in the unconfirmed TB patients in unavailable, all children in this study group demonstrated improvement of symptoms after completion of TB treatment. While this is preliminary data, the breathprint approach could be appealing as a clinically-relevant diagnostic tool for pediatric patients, especially to distinguish those with TB who have unconfirmed TB.

Previously, Zar and colleagues demonstrated an improvement in sensitivity of Ultra for culture confirmed TB disease in children by testing multiple samples for Ultra; a single induced sputum (sensitivity of 74.3%), two nasopharyngeal aspirates (sensitivity of an individual test is 46%) or combination of sputum and nasopharyngeal samples providing an overall sensitivity of 87.5%^4^. Given the 4-compound breathprint’s sensitivity to both confirmed and unconfirmed pediatric TB cases, using it as a triage test prior to Ultra testing may further increase sensitivity in confirmed TB patients while adding further diagnostic evidence for unconfirmed TB patients.

These results, while positive, have limitations. The 4-compound breathprint may only applicable to mixed expiratory fixed-volume sampling method with patients breathing normally. Further evaluation will be needed if different breath sampling methods are used or different patient breathing patterns are employed, as some breath VOCs have been reported to be dependent on exhalation flow and the portion of the breath collected^59–64^. In addition, exhalation flow monitoring was not possible due to the design of our sampling kits. Sampling device with flow monitoring capabilities are currently under development in our laboratory. Further evaluation will be conducted when the flow monitoring sampling devices become available.

As a multi-center breath-analysis study, the effect of transportation and storage has always been a concern for breath samples using sorbent tubes, especially when no specific guideline has been established by European Respiratory Society (ERS)^65^. Other studies have indicated that the stability of breath compounds varies and may depend on sampling media (sorbent material), storage temperature and time, and the breath compositions^66–71^. Some molecules such as benzene, toluene and m-xylene are stable for 12 months on Tenax TA TD tubes^67^, but in general, researchers suggested that analysis by day 14 in cold storage will minimize a potential 1–2 standard deviation gain or loss of VOC concentration^71^. For this and many other multi-center studies, sample analysis within 14 days of collection is usually not feasible. Integration of stability tests for novel breath molecules in the current biomarker discovery study is even more challenging. Therefore, future independent studies on the transportation and storage stability of the 4-compound breathprint are required to ultimately validate this result.

While assessing performance statistics across cross validation folds gives a more accurate indication of generalization than the final model, it has been suggested that estimates originating from cross validation may still be overly optimistic^72^. Due to the pilot nature of this study, validation of these results across a larger sample is necessary. Indeed, a larger population would allow assessment of additional co-morbidities (such as diabetes, childhood asthma, and more robust analyses in HIV + children), spectrum of TB disease, and other population characteristics that could influence the predictive ability of the TB pediatric breathprint. Moreover, this study cannot conclude if these results will generalize to populations outside of South Africa. Future work should consider a multi-site study aimed at evaluating breath as a diagnostic medium for pediatric TB across many endemic countries. Furthermore, while the unconfirmed TB group had clinical symptoms and chest radiographs suggestive of TB disease, microbiological confirmation was negative. Although unconfirmed patients improved while undergoing TB treatment, a gold standard diagnosis of TB is not possible in this group. Finally, the study is underpowered to confidently propose the 4-compound breathprint and subsequent random forest model as clinical instruments to diagnose TB in children. However, we confidently conclude that breath as a medium for diagnosis of pulmonary TB in pediatric patients in conjunction with machine learning models is feasible, demonstrates clinical utility, and warrants further investigation.

## Methods

### Study subjects and design

Study subjects were recruited, diagnosed, and treated in a prospective clinical study described previously^4^. In short, consecutive children hospitalized between April 4th 2017 and December 14, 2017 in Cape Town, South Africa with suspected TB were enrolled. Study eligibility criteria were age less than 15 years, cough of any duration, and at least one of the following: a household TB contact within the previous 6 months, weight loss or failure to gain weight within the previous 3 months, a positive tuberculin skin test or a chest radiograph suggesting pulmonary TB. All children had a chest radiograph, a tuberculin skin test if there was no known previous TB diagnosis, and HIV testing when HIV status was unknown. TB therapy was initiated at the discretion of the treating doctor. Response to treatment was assessed at follow up at 1, 3 and 6 months by recording signs and symptoms.

Children were classified according to diagnostic categories: ‘confirmed TB’ (culture or Xpert positive for *Mtb*), unconfirmed TB’ (microbiologically negative, clinically diagnosed) or ‘unlikely TB’ (microbiologically negative, not clinically diagnosed, no tuberculosis treatment given, and documented improvement at follow up).

The Research Ethics Committee of the Faculty of Health Sciences, University of Cape Town (#045/2008) and the Committee for the Protection of Human Subjects at Dartmouth College approved the study (STUDY00030329). All methods were performed in accordance with relevant guidelines and regulations and identifying information is not presented in this report. Informed consent was obtained through parents or legal guardians.

### Breath collection kits and procedure

A mixed expiratory fixed-volume sampling method was used, following the guidelines from European Respiratory Society (ERS) technical standard for exhaled biomarkers in lung disease^65^. Mixed expiratory breath and room air samples were collected using kits and protocols at the time of study enrollment as described previously^12^. In short, kits consist of a 1.5L Tedlar bags with a drinking straw mouthpiece for patients to breath into. Patients rinse mouth with water, and then are asked to breathe normally into the bag until it is full. Breath is then drawn through a 13 mm, 0.22 µm PTFE filter and into 3-bed thermal desorption tubes (TDT), using a vacuum pump. All samples were collected at time of enrollment, prior to commencement of treatment. Samples were shipped from Cape Town South Africa to Hanover, New Hampshire, United States of America and stored at 4 °C. Samples were processed within 6 months of collection.

### Analytical instrumentation and initial processing

The breath compounds were collected on the TDT and desorbed at 330 °C into a cryogenically cooled (-120 °C) inlet liner of a GC × GC-TOFMS instrument (LECO Corporation, MI, USA). After desorption, the inlet is rapidly heated from − 120 to 270 °C and the trapped breath compounds are transferred onto an Rxi-624Sil-MS/Stabilwax chromatography columns. The TOFMS collected spectra over the range of *m*/*z* 30–500 at a rate of 200 Hz. For peak findings, a signal-to-noise (S/N) cutoff was set at 50:1 (with a minimum of three apexing masses) in at least one chromatogram and a minimum of 20:1 S/N in all others. The NIST 11 library was used for the initial identification of the analytes. A chemical formula was assigned if the analytes matched the following three criteria, (1) high mass spectral match, (2) group separation based on the structural formula and (3) the EIC ionization patterns among all observed samples. To verify the chemical formulas of discriminatory features, authentic standards were purchased, spiked into blank thermal desorption tubes, and run using the same analytical method as the breath samples. Retention indices were determined using C8–C20 n-alkane standard solution for both sample runs and standard runs. If both mass spectra and retention index of a feature is matched with the standard, the chemical structure the feature is confirmed. Alkane Standard Solution C8-C20 (~ 40 mg/L each in hexane) was purchased from Supelco (Darmstadt, Germany) and stored at 4 °C. 4-Mehyloctane was purchased from Toronto Research Chemicals (North York, ON, Canada,) and stored at 4 °C. The analytes that were not given a formula did not match on any of the previous criteria. Possible contaminants are manually removed before further data analysis (see Supplementary Table 6 for details).

### Statistical analysis

A brief summary of our data cleaning and feature reduction process is shown in Supplementary Fig. 5. All statistical analyses were conducted in R 3.6.1 (R Core Team, Vienna, Austria)^73^. Data cleaning was followed as described previously^12^. In short, a frequency of observation (FOO) cutoff of 80% in either the confirmed TB or unlikely TB categories was implemented. Remaining features were normalized using PQN, log_10_ transformed, and mean centered. Missing values were imputed using a random forest imputation^74^. Features were further reduced using a Mann–Whitney U-test to find features that were significantly different between patients and room air (Benjamini–Hochberg adjusted *p* value < 0.05)^75,76^. A Boruta feature selection scheme was then used to find features which could discriminate between confirmed TB and unlikely TB groups^77,78^. It is recommended that pilot studies employ a more forgiving statistical threshold given that they are underpowered and designed for exploratory rather than confirmatory analysis. It is often recommended that pilot studies report findings as significant at a 75–85% confidence level and do not adjust for multiple comparisons^79,80^. Here, we consider a significance level of $\alpha$=0.1 for statistical significance of the selected features to balance the pilot nature of this work while remaining appropriately conservative for follow-up studies.

After features were selected, models were built using a fivefold cross validation (CV) scheme in the ‘caret’ package^81,82^. CV splits the data into 5 equal size pieces, builds a model on 4 of the five pieces, and tests it on the remaining piece. It then leaves a different piece out and repeats this process^82^. This allows for parameter tuning across the models, as well as gives an estimate of model generalizability by examining accuracy statistics across the left-out pieces. All performance statistics are reported based on their performance across validation folds as these are more representative of performance and less influenced by overfitting^72^. Many models are sensitive to class imbalance, so an up-sampling scheme was used to split the data^81^.

We fit two models on the data, random forest and a polynomial support vector machine^43,83^. Random forest models build a ‘forest’ of ‘decision trees’ where features are selected randomly in each tree according to how well they split the data^83^. Polynomial support vector machines fit a polynomial hyperplane between groups of interest in n-dimensional space^43^. Both models were built to classify between confirmed and unlikely TB patients and then used to predict the TB status of unconfirmed TB patients.

## Supplementary Information

Supplementary Tables.

Supplementary Figures.

## Data Availability

The datasets generated during and/or analyzed in the current study are available from the corresponding author on reasonable request.
