# Supplementary material for: Breath can discriminate tuberculosis from other lower respiratory illness in children
Source: Sci Rep. 2021 Feb 1;11:2704. doi: 10.1038/s41598-021-80970-w (PMC7851130; doi:10.1038/s41598-021-80970-w)
Supplement: Supplementary file 2 — Supplementary Figures. [file 41598_2021_80970_MOESM2_ESM.pdf]

Breath can discriminate tuberculosis from other lower respiratory illness in children

Carly A. Bobak, Lili Kang[§], Lesley Workman[§], Lindy Bateman, Mohammad S. Khan, Margaretha Prins, Lloyd May, Flavio A. Franchina, Cynthia Baard, Mark P Nicol, Heather J. Zar[‡], Jane E. Hill[‡]*

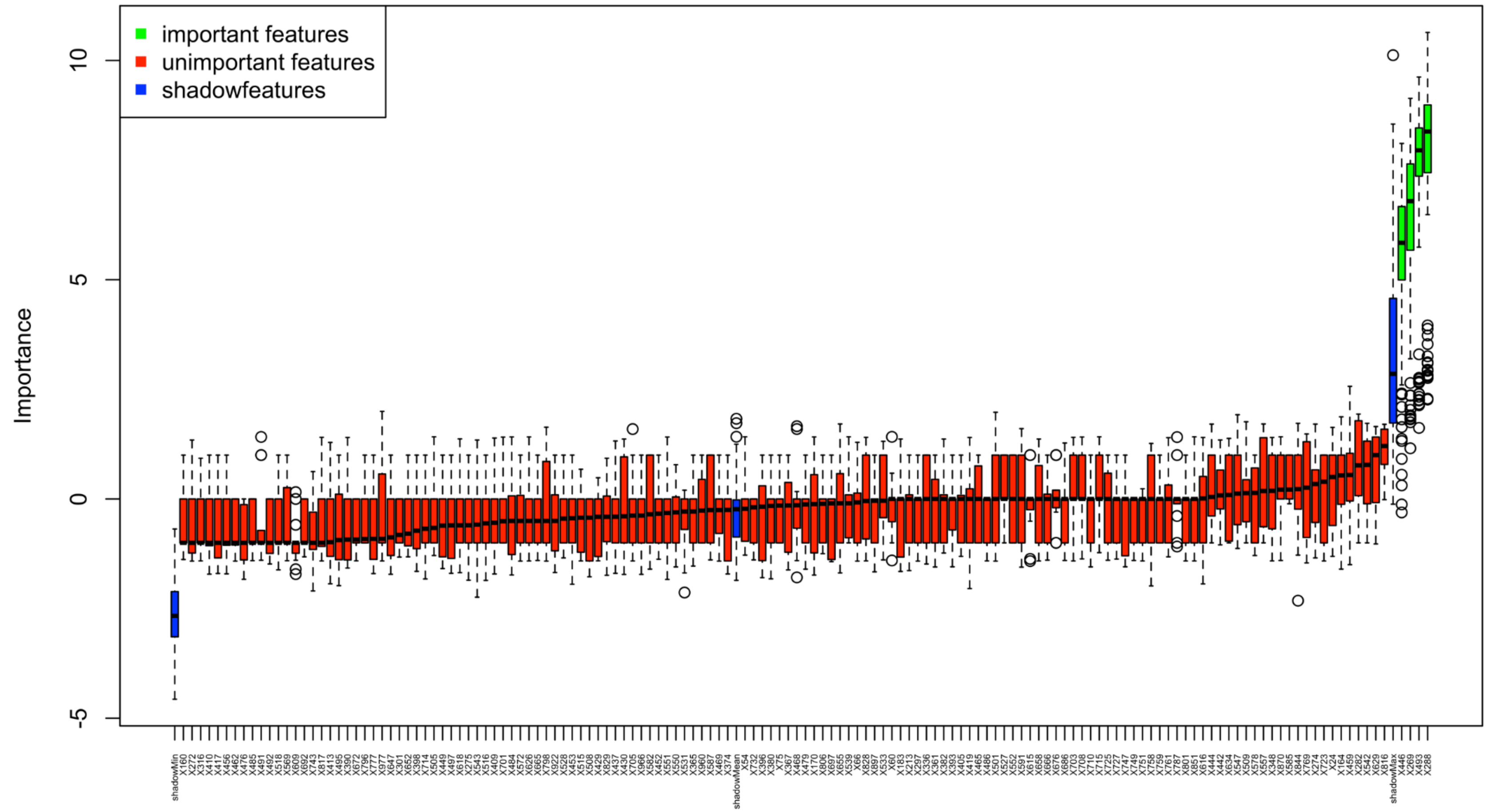

Supplementary Figure 1: The results from the Boruta feature selection algorithm over 84 iterations, at which point Importance had converged. The blue boxplots represent the range of observed importance values of the shadow variables (permutations of the observed data), the red boxplots are the unimportant features, the green boxplots are the selected important features. Figure made in R[73]

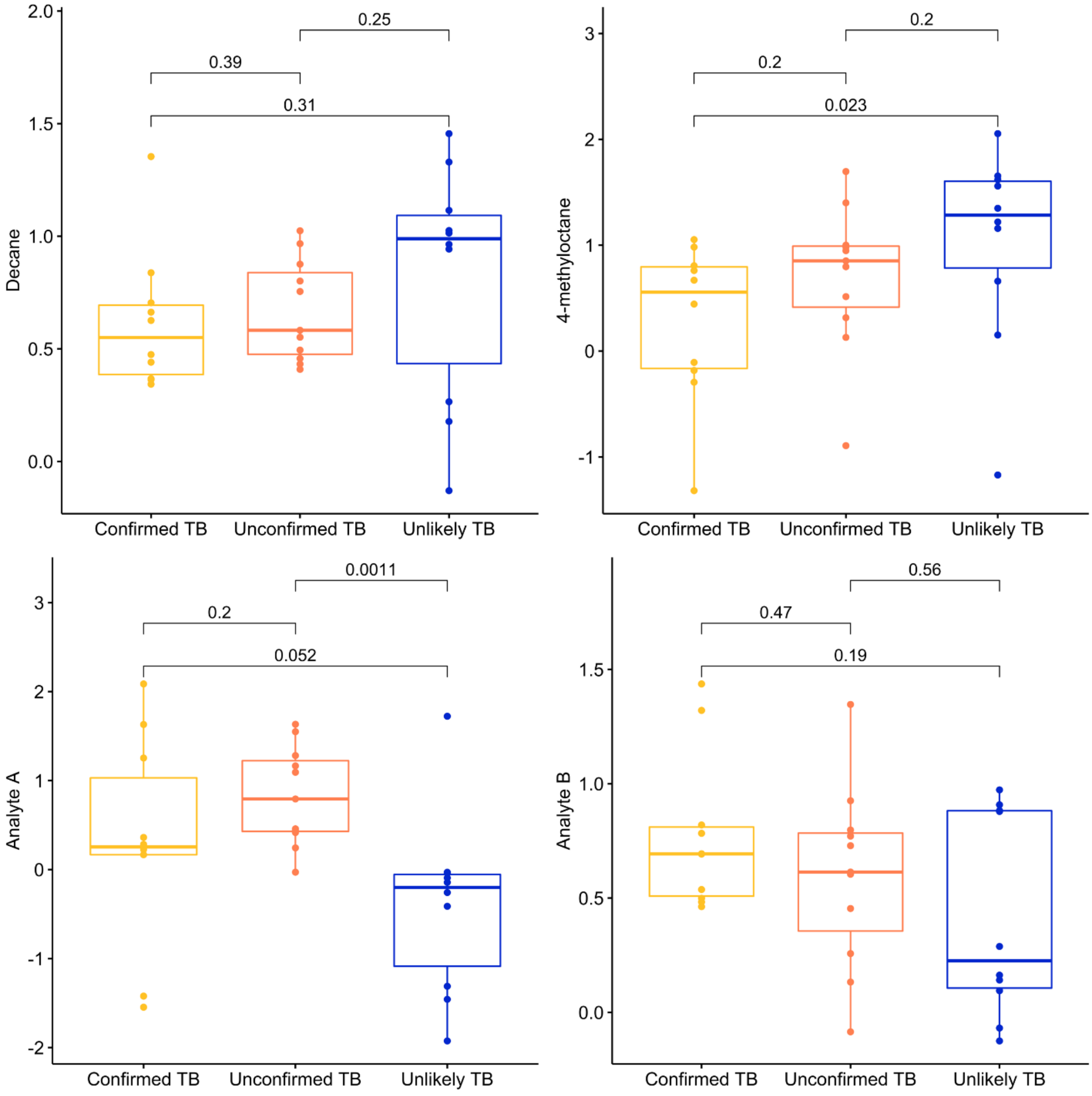

Supplementary Figure 2: The distribution of the normalized peak area of each of the four compounds selected in the breathprint across confirmed TB, unconfirmed TB, and unlikely TB patients. No compound was statistically significantly different between confirmed TB and unconfirmed TB patients, where Analyte A was statistically significant between confirmed TB and unlikely TB as well as unconfirmed TB and unlikely TB. While statistically significance was not reached in many cases, the distribution of the centered and normalized peak area appears to be far more similar across confirmed/unconfirmed TB patients as opposed to unlikely TB patients. Increased sample size may make these differences more apparent. Figure created in R[73] using ‘ggplot2’[82] and ‘ggpubr’[83].

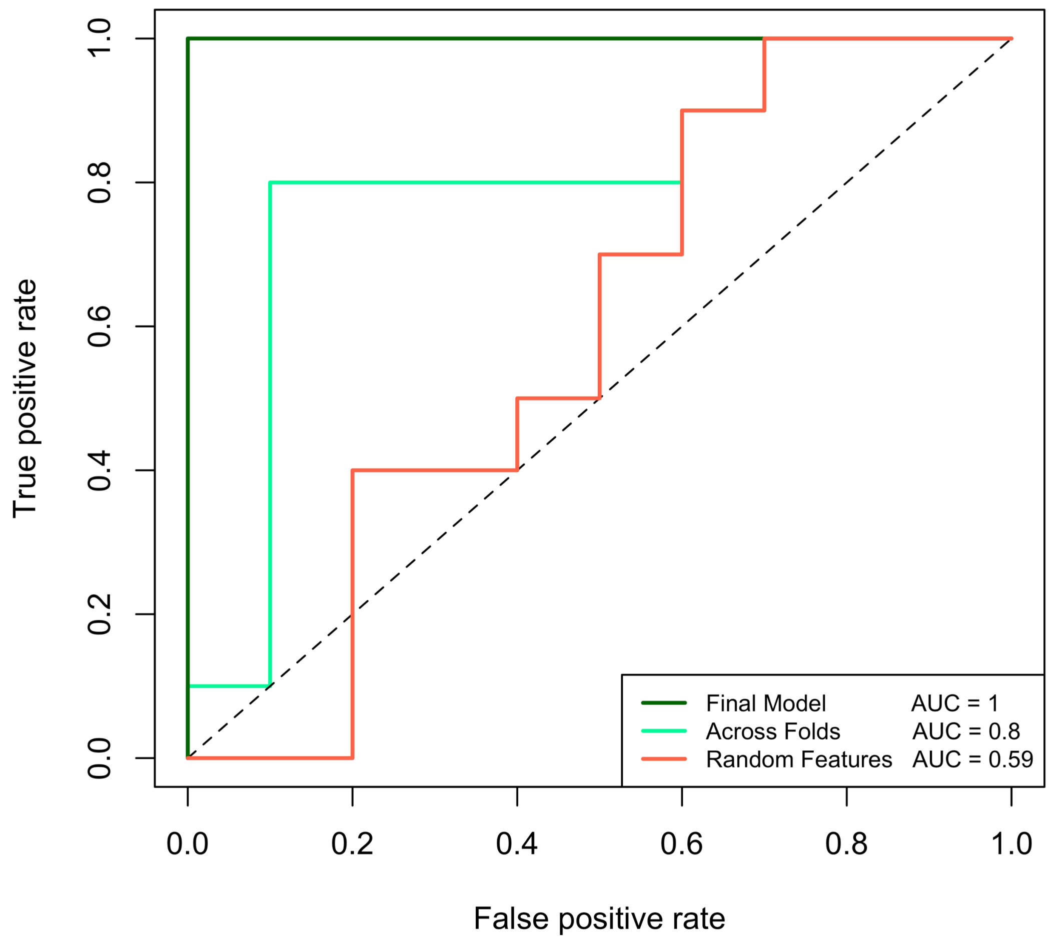

Supplementary Figure 3: The ROC curves from the polynomial SVM model. While the final model demonstrated perfect performance, the AUC observed across folds can be used as an indication of the generalizability of results to independent data. The random feature line represents the AUC observed across folds of the algorithm if four random compounds are selected to build a polynomial SVM model. Figure created in R[73].

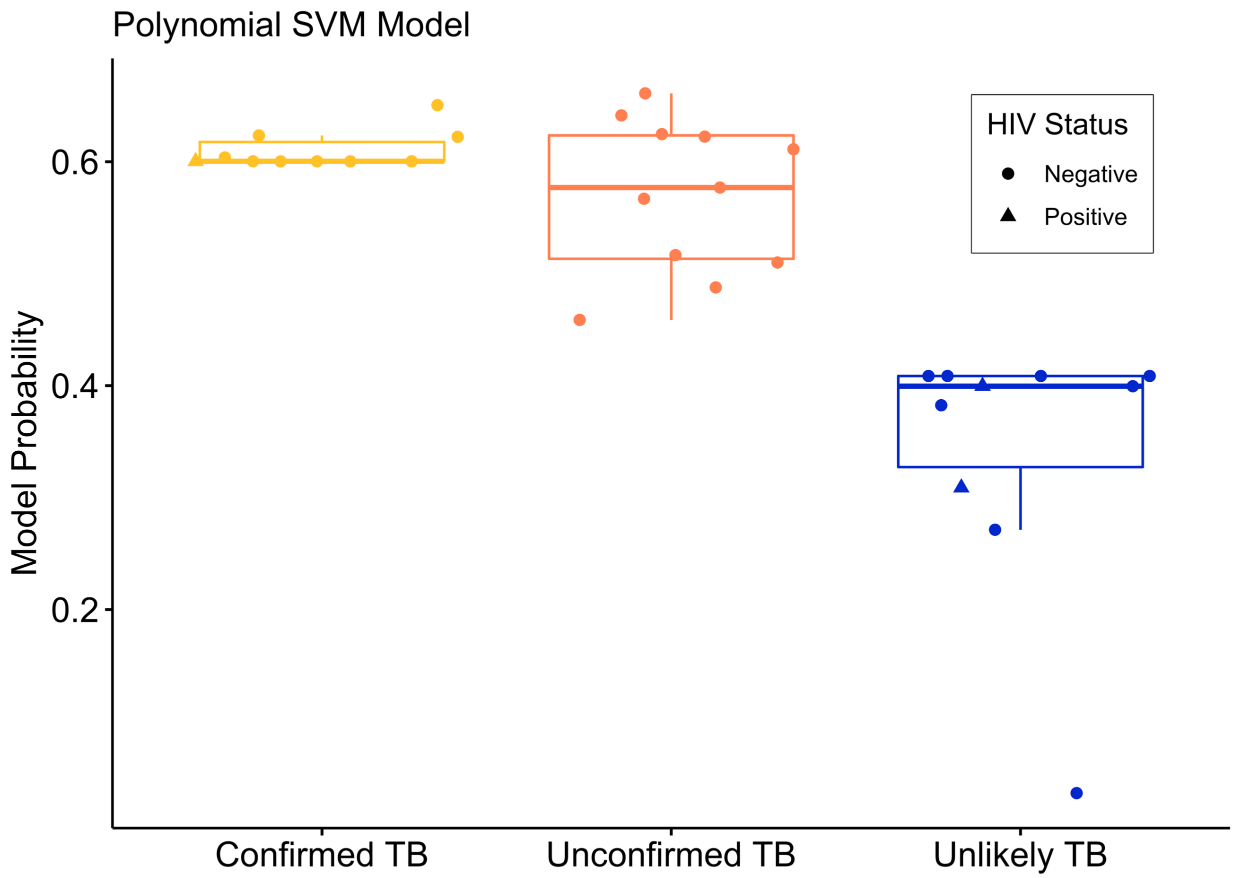

Supplementary Figure 4: Model probabilities from the SVM classifier across the TB categories. The higher the model probability, the more likely the classifier is to label the case as having TB disease. The confirmed TB an unconfirmed TB groups have overlapping probabilities, indicating that for many patients the classifier works well despite culture confirmation. While some of the unconfirmed TB patients have a probability of less than 50% for having TB disease, there is clear differentiation between the scores of unconfirmed TB patients and unlikely TB patients. Figure created in R[73] using ‘ggplot2’[82] and ‘ggpubr’[83].

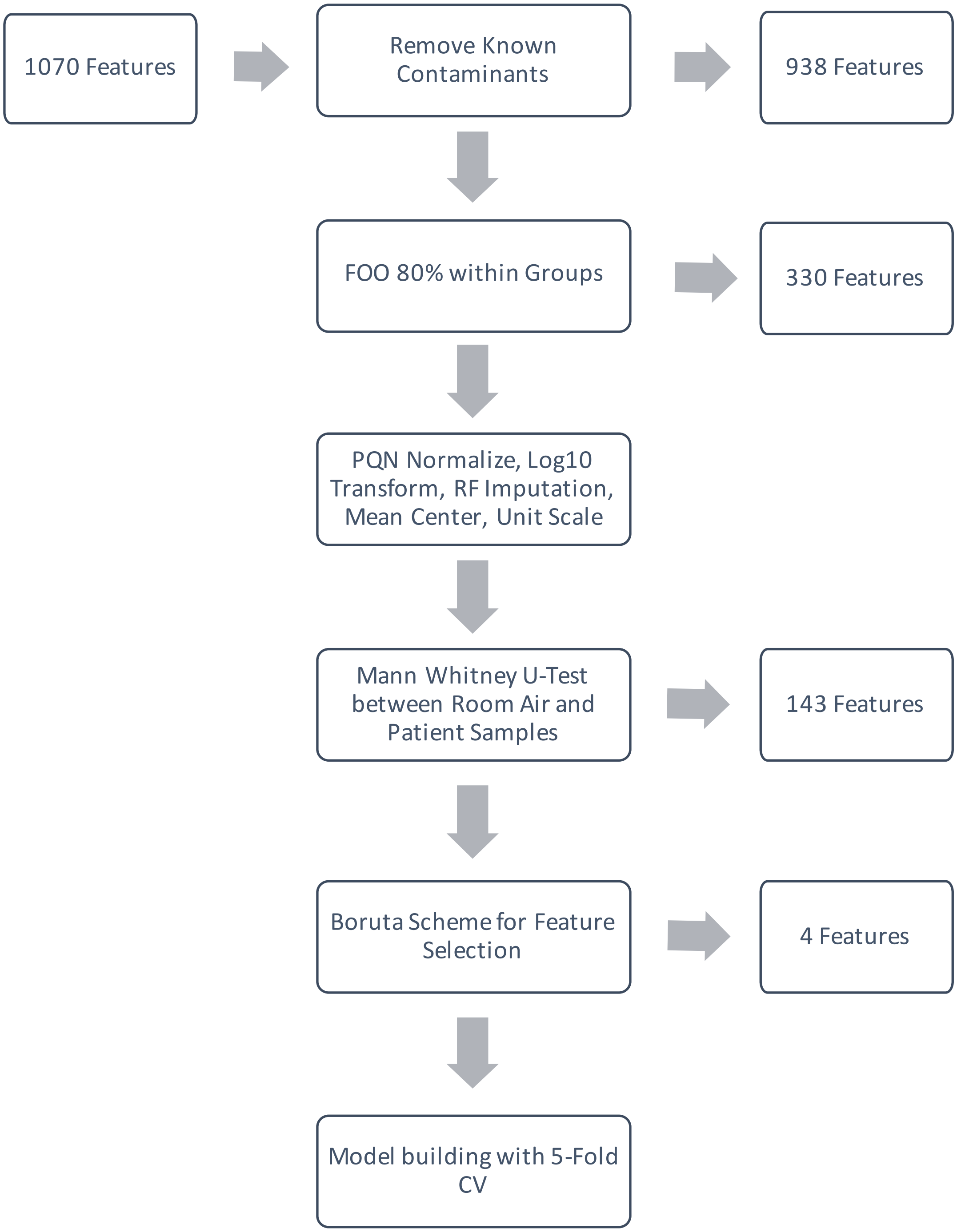

Supplementary Figure 5: The statistical analysis and feature reduction strategy used to identify the 4-compound breath print

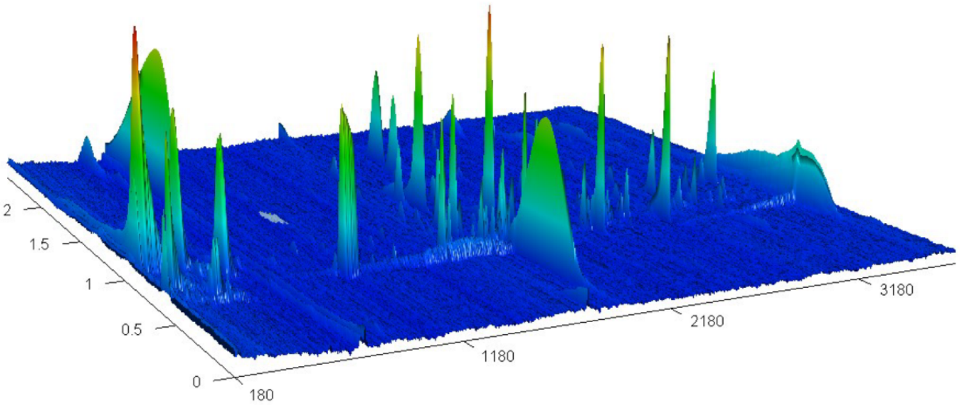

Supplementary Figure 6: Representative 2-dimensional chromatogram of a breath sample

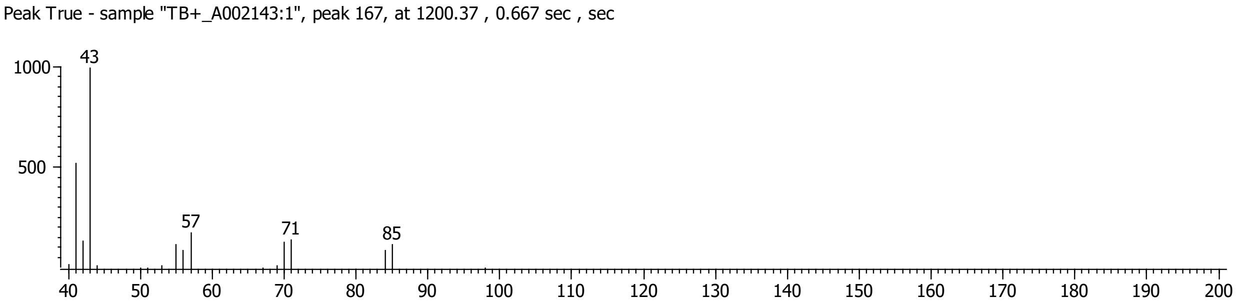

Supplementary Figure 7: Representative mass spectra of 4-methyl- octane detected in a breath sample

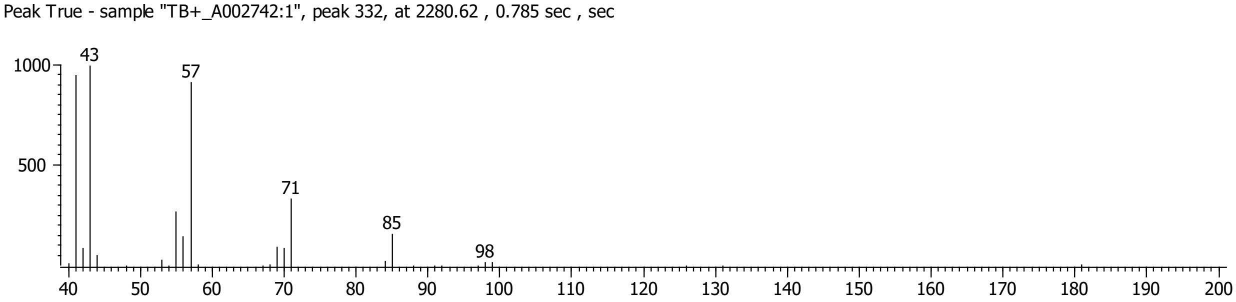

Supplementary Figure 8: Representative mass spectra of decane detected in a breath sample

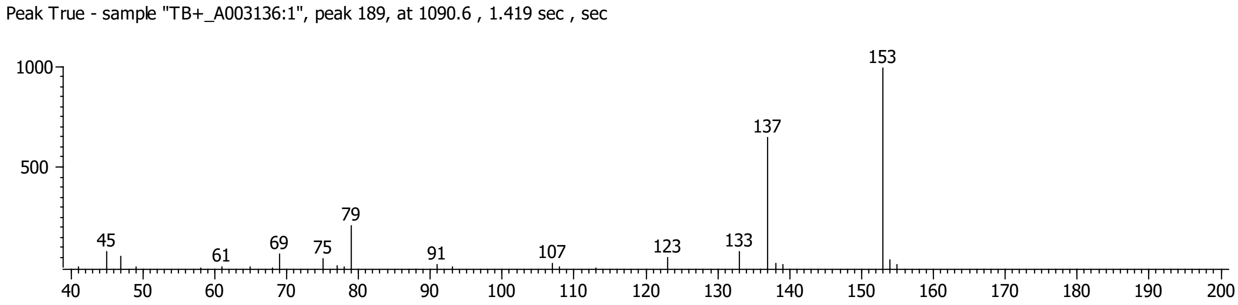

Supplementary Figure 9: Representative mass spectra of Analyte A detected in a breath sample. An odd m/z 153 peak suggests it contains nitrogen. The high intensity of this peak indicates it is stable and likely to be a cyclic compound. A loss of 16 from m/z 153 to 137 is likely due to the loss of NH_2 from a benzamide. Peaks at m/z 79, 91 and 107 also suggest it has a benzene ring.

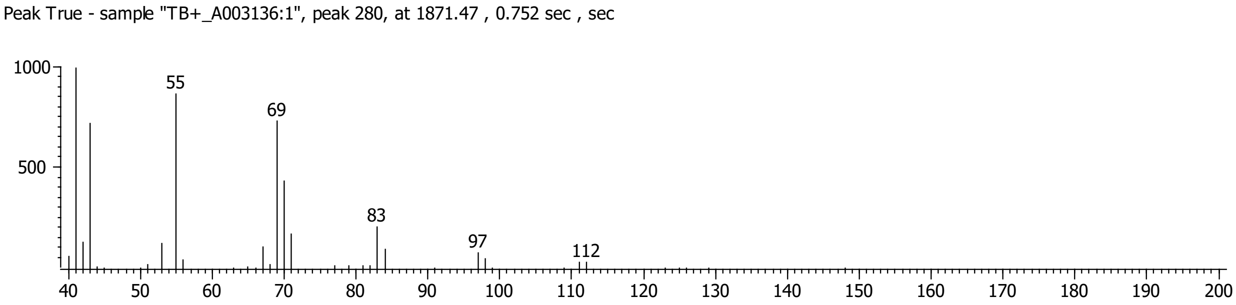

Supplementary Figure 10: Representative mass spectra of Analyte B detected in a breath sample. The m/z 55, 69, 83 and 97 peaks correspond to C_4H_7, C_5H_9, C_6H_{11} and C_7H_{13} fragments of an alkene. The lack of decay of the peak intensities at m/z 55 and 69 indicate a favored C_4 and C_5 fragment, suggesting a methyl group on third or fourth carbon. It has 11 carbons based on its retention index.
